# Residual *β*-Cell Function in Type 1 Diabetes Followed for 2 Years after 3C Study

**DOI:** 10.1155/2021/9946874

**Published:** 2021-06-28

**Authors:** Kun Lin, Xiaoping Yang, Yixi Wu, Shuru Chen, Qiong Zeng

**Affiliations:** ^1^Department of Endocrinology, The First Affiliated Hospital of Shantou University Medical College, Shantou, China; ^2^Shenzhen Huada Gene Technology Service Co., Ltd, Shenzhen, China; ^3^Department of Neurology, The First Affiliated Hospital of Shantou University Medical College, Shantou, China

## Abstract

**Objective:**

To investigate the natural history and related factors of the pancreatic *β*-cell function in Chinese type 1 diabetic patients from 3C study Shantou center.

**Method:**

Stimulated C-peptide levels from follow-up data of 201 individuals in 3C study Shantou subgroup starting in 2012 were used. Residual *β*-cell function was defined as stimulated C − peptide level ≥ 0.2 pmol/mL, on the basis of cut-points derived from the Diabetes Control and Complications Trial (DCCT).

**Results:**

36.8% of patients had residual *β*-cell function, and the percentage was 68.2% in newly diagnosed diabetic patients. COX regression analysis indicated that the age of diagnosis, HbA1C level, and duration were independent factors of residual *β*-cell function in individuals with ≤5 years duration, but in those with duration ≥5 years, only the age of diagnosis was a predictor. The pancreatic *β*-cell function mainly declined in the first 5 years of the duration, and the rate of decline was correlated negatively with the duration and age of diagnosis. Receiver operating characteristic (ROC) analysis indicated that the cut-off point of stimulated C-peptide was 0.615 pmol/mL in patients with <5 years duration to have 7% HbA1c.

**Conclusion:**

Age at diagnosis was the strongest predictor for residual C-peptide. There was a more rapid decline of stimulated C-peptide in duration ≤5 years and younger patients. Therefore, intervention therapies of *β*-cells should start from the early stage, and the recommended target goal of stimulated C-peptide is 0.615 pmol/mL or above.

## 1. Background

Type 1 diabetes mellitus (T1DM) is an autoimmune disease with severe pancreatic *β*-cell deficiency. Traditionally, it was considered that the *β*-cell function declined quickly over time from the onset, and the extremely low C-peptide level had little effect on metabolism [1]. But now, it is challenged. The Diabetes Control and Complications Trial (DCCT) showed that T1DM patients with residual *β*-cell function (stimulated C − peptide ≥ 0.2 pmol/mL) had better metabolic control and lower rates of complications compared to those with C-peptide levels below this threshold [2, 3]. The Joslin 50-year medalist study found that most of the T1DM participants with a duration of 50 years or more reached endogenous clinical insulin secretion (random C − peptide ≥ 0.03 pmol/mL) [4], and 2.6% had random serum C − peptide ≥ 0.20 pmol/mL. Recently, the pancreatic *β*-cell function in T1DM had been investigated in the TrialNet Study [5], Search Study [6], and European multicenter clinical study [7]. However, the natural history of *β*-cell function is still ambiguity, especially in long-duration patients, and the related factors of the decline of *β*-cell function are controversial. In T1DM patients, the C-peptide level with clinical significance and its influence on glycemic control are still unknown.

In recent years, the assessment of *β*-cell function has received more and more attention. The rise of stem cell therapy, immunotherapy presses for an in-depth and clear understanding of the natural course of *β*-cell function in T1DM. Therefore, translational medicine is necessary to provide more evidence of the timing choice for the intervention, the inclusion criteria, and the curative effect evaluation, even clinical researches and designs in the future.

The 3C study was launched in 2011-2012 by International Diabetes Federation (IDF) [8]. As a milestone in the history of Chinese T1DM researches, the 3C study is by far the largest Chinese T1DM clinical study to investigate the coverage, cost, and care of T1DM in China [9–11]. Individuals of the 3C study Shantou subgroup had been followed up since 2012. Follow-up data including *β*-cell functions was analyzed in order to discuss the previous related issues and have a deeper understanding.

## 2. Methods

### 2.1. Subjects

Data derived from the 3C study was described previously [8]. The IDF selected health care facilities for the study from primary (four Beijing; two Shantou), secondary (three Beijing; two Shantou), and tertiary (six Beijing; two Shantou) levels of care that had active diabetes outpatient clinics and the willingness and capacity to participate in the study. Participants were recruited from Shantou 3C center (including six facilities above) and had been followed up for 2 years since 2012 when 3C study finished. For those the age of diagnosis over 35, the diagnosis of T1DM was confirmed in the main questionnaire by recorded deficient C-peptide levels, repeated ketosis/ketoacidosis and/or uncontrolled hyperglycemia during the first six months of diabetes, or history of ketosis/ketoacidosis within 6 months of initial diagnosis with therapy of oral hypoglycaemics drugs or without insulin. Those diagnosed younger than 6 months were also excluded [12].

### 2.2. Measurements

Participants underwent follow-up every 3 months for 2 years (from April 2012 to April 2014). Each follow-up was defined as visit 1 (V1), visit 2 (V2), visit 3 (V3), visit 4 (V4), visit 5 (V5), visit 6 (V6), visit 7 (V7), and visit 8 (V8) in chronological order. Demographic data, types, and doses of insulin and oral hypoglycaemics drugs, physical examination, and clinical laboratory reports were recorded in case report forms of every follow-up.

### 2.3. Laboratory Analyses

Participants received laboratory tests regularly. HbA1c was measured quarterly (high-pressure liquid chromatography), urine ACR was measured half a year, and fundus photography and blood lipids were assessed annually. Pancreatic autoimmune antibodies (GAD, ICA512, and ZnT8) were determined at the onset. Basal and stimulated plasma C-peptide concentrations (ELISA) were measured annually (at V0, V4, and V8). 70 g instant noodles were used for the standardized meal stimulation test. Cut point of residual *β*-cell function was defined as stimulated C − peptide ≥ 0.2 pmol/mL, based on the Diabetes Control and Complications Trial (DCCT).

### 2.4. Statistics

Data were analyzed by statistical software SPSS Version 17 for Windows. According to the *β*-cell function in the baseline, participants were divided into 2 groups, with residual *β*-cell function (stimulated C − peptide ≥ 0.2 pmol/mL) and not. One-way ANOVA was used for unequal variances and *χ*^2^-test for categorical variables between groups. The COX regression model was used to test associations between independent variables of interest and residual *β*-cell function status during follow-up. The following independent variables were included into the COX regression models: age, gender, the age of diagnosis, body mass index (BMI), presence or absence of diabetic ketoacidosis (DKA) at the onset, family history of diabetes, HbA1c at diagnosis, mean HbA1c, and the numbers of positive autoantibodies. Statistically significant variables were included in a forward stepwise approach. Furthermore, we analyzed a cut-off value of stimulated C-peptide level by receiver operating characteristic (ROC) analysis, in order to predict whether individuals reached HbA1c < 7% or not. *P* value < 0.05 was considered statistical significance.

## 3. Results

240 patients with T1DM were enrolled, and 201 finished 2-year follow-up. The clinical data were shown in Table 1. At baseline, the median age was 24.77 years, median age at onset 18.72 years, and median duration of T1DM 5.12 years. Proportions of duration <1 year, 1-5 years, 5-10 years, and >10 years were 11%, 38%, 36%, and 15%, respectively. HbA1c was 10.04 ± 2.90%. BMI was 18.36 ± 4.73 kg/m^2^.

36.8% patients had residual *β*-cell function, and in the newly diagnosed patients, the percentage was 68.2%. Compared to those without residual *β*-cell function, patients with residual *β*-cell function had older ages of diagnosis, shorter durations, and lower HbA1c levels. There were no significant differences in gender, age, BMI, numbers of positive antibodies, blood lipids, and prevalence of diabetic complications (including hypoglycemia, retinopathy, microalbuminuria, and proteinuria) between both groups.  Table 2 presented the results of the COX regression models with the dichotomous outcome of residual C-peptide status. Variables independently associated with residual *β*-cell function in individuals with duration ≤5 years included older age of diagnosis, lower mean HbA1C level, and shorter duration. With regard to those with a duration of ≥5 years, only age of the onset was related.

**Figure 1:**
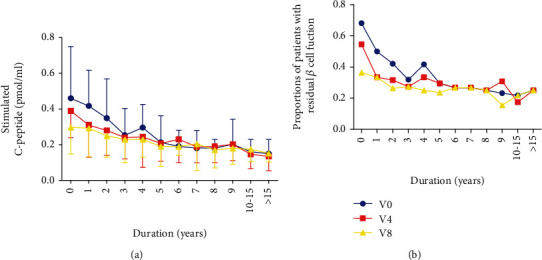
*β*-cell function by duration of diabetes. Changes of C-peptide in T1DM with different diabetes duration during different visits (a). Changes of proportion of individuals with residual *β*-cell function during different visits (b). In the individuals with duration < 5 years, stimulated C-peptide at V0 was significantly different with C-peptide at V4 (*P* < 0.05) and V8 (*P* < 0.05), the proportion of individuals with residual *β*-cell function at V0 was significantly higher than that V4 (*P* < 0.05) and V8 (*P* < 0.05). Among those with duration ≥ 5 years, there were no significant differences in stimulated C-peptide levels among them. V0: the 0th visit; V4: the 4th visit; V8: the 8th visit.

**Figure 2:**
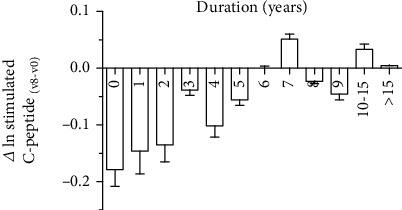
Decline of stimulated C-peptide over 2 years (from v0 to v8). The difference of C-peptide between v8 and v0 by self-comparison in T1DM with different diabetes duration was processed with logarithm in order to reflect the changes of islet *β*-cell function intuitively. Among those with a duration of ≤5 years, a log-linear decline of stimulated C-peptide was observed during individuals with diabetes duration 0 to 5 years (*P* < 0.01). However, among those with a duration of ≥5 years, changes of stimulated C-peptide levels were irregular. V0: the 0th visit; V8: the 8th visit.

Furthermore, the relationship between diabetes duration and *β*-cell function was analyzed. The C-peptide levels and the proportion of individuals with residual *β*-cell function diminishing with increasing durations were shown in Figures 1(a) and 1(b), respectively. Pancreatic *β*-cell functions were preserved better in patients with shorter durations than those with long-durations, but significant differences were only presented in duration <5 years. Levels of stimulated C-peptide gradually declined from diagnosis to duration of 5 years. Among those with duration of ≥5 years, there were no significant differences in stimulated C-peptide levels among them. The decline degree of residual *β*-cell function was analyzed using stimulated C-peptide levels over 2 years (v8-v0) by self-control. The decline in individuals with a duration of ≤5 years was significantly faster than those ≥5 years. Among those with a duration of ≤5 years, a log-linear decline of stimulated C-peptide was observed from diagnosis to the duration of 5 years (Figure 2). However, among those with a duration of ≥5 years, changes of stimulated C-peptide levels were irregular.

**Figure 3:**
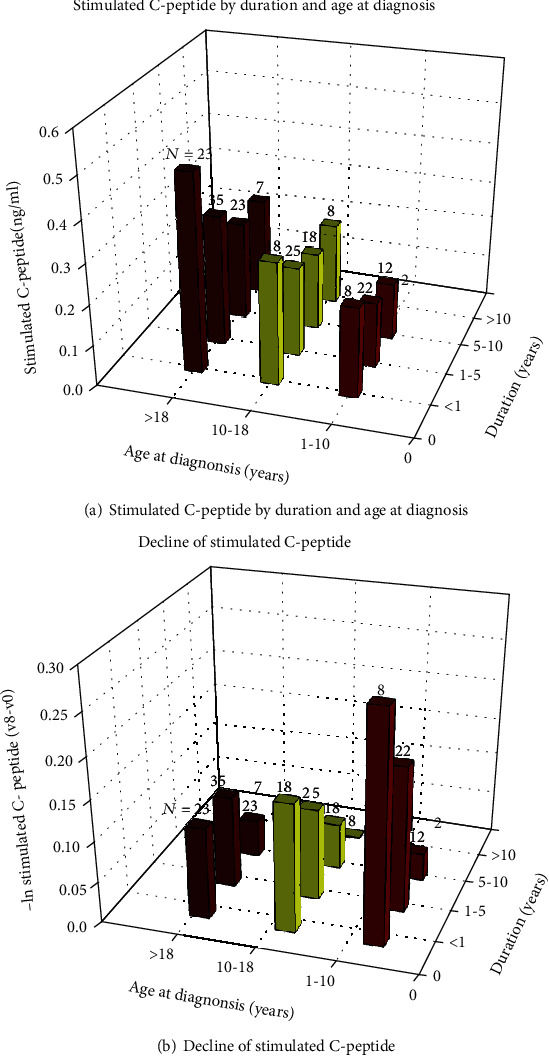
Stimulated C-peptide by duration and age at diagnosis. The associations of age at diagnosis and diabetes duration with stimulated C-peptide were illustrated in (a). Each box represents the mean stimulated C-peptide of the individuals under the corresponding condition of diabetes duration and age of diagnosis. The associations of age at diagnosis and diabetes duration with decline of stimulated C-peptide (v8-v0) were shown in (b). Each box represents the mean decline of stimulated C-peptide (v8-v0) under the corresponding condition of diabetes duration and age of diagnosis. V0: the 0th visit; V8: the 8th visit.

The associations of age at diagnosis and diabetes duration with stimulated C-peptide were illustrated in Figure 3. Individuals with younger age at diagnosis, longer diabetes duration had lower levels of stimulated C-peptide and faster decline rates. The positive correlation between age at diagnosis and stimulated C-peptide was present in all duration groups. However, the negative correlation between diabetes duration and stimulated C-peptide level was shown only in individuals with diabetes duration ≤5 years.

**Figure 4:**
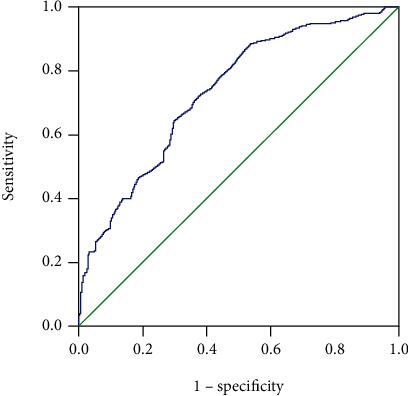
Receiver operating characteristic curve analysis of stimulated C-peptide cut point for HbA1c ≤ 7.0%. Area under curve = 0.733 (*P* < 0.001), 95% confidence interval: 0.673–0.793; identified cystatin C cut point = 0.615 ng/mL; sensitivity: 72.63%; specificity: 61.77%.

Finally, the relation between stimulated C-peptide level and mean HbA1c each year in individuals with duration of ≤5 years was analyzed. C-peptide and HbA1c scatter perfectly fitted a logarithmic curve, *P* < 0.001. ROC curve analysis was performed to verify the prediction accuracy of stimulated C-peptide level for HbA1c, using the HbA1c threshold value of 7% as outcome variable. The area under curve was 0.733 (95% confidence interval 0.673-0.793), and the optimal cut point of the C-peptide level was 0.615 pmol/mL (Figure 4). The sensitivity was 72.63%, and specificity was 61.77% at this cut point.

## 4. Discussion

The study indicated several different clinical characteristics in China's T1DM patients compared with the Caucasians: the later onset age, lower BMI, and higher levels of HbA1c [13–16]. DCCT suggested stimulated C − peptide ≥ 0.2 pmol/mL as the indicator of residual *β*-cell function. In this study, according to this reference standard, 36.8% of the patients retained residual *β*-cell function, and the percentage was 68.2% in the newly established patients. Similar to our results, many studies had demonstrated that certain *β*-cell function was detectable in various courses of T1DM. DCCT showed that, with duration < 5 years, 33% of subjects with age < 18 years had stimulated C − peptide levels ≥ 0.2 pmol/mL, and the percentage was 48% in those with age >18 years [1, 3]. In the Search Study, investigated young American diabetic patients with positive autoantibodies, up to 82.9% of patients preserved fasting C − peptide ≥ 0.2 pmol/mL within 1 year after the onset [16]. Whereas, only 69% of newly diagnosed T1DM patients retained fasting C − peptide ≥ 0.07 pmol/mL within the first year of duration in the European multicenter clinical study [7]. 67.4% of the Medalists in the Joslin 50-year medalist study reserved residual beta-cell function [4], the proportion was 46% in subjects with duration ≥ 25 years in Keenan et al.'s study [17], 40% in individuals with 10-30 years of duration in Pipeleers' research [18], 88% in patients with 4-67 years of T1DM in Meier et al.'s study [19], and 13% in patients with 10 years T1DM in Gianani et al.'s study, respectively [20].

In this study, the proportion of patients with residual *β*-cell function was lower than that of Caucasians in previous studies. Similarly, Taiwanese children with T1DM had low fasting and stimulated C-peptide levels at diagnosis [21]. The ethnic difference may explain partially the variation of the pancreatic *β*-cell function in T1DM. But it was more likely that the difference of glycemic control between Chinese and Caucasians played an important role in the pancreatic *β*-cell function preservation. Compared to Caucasians, poorer glycemic control and higher HbA1c level in Chinese patients were due to the lack of early appropriate interventions and managements found in the 3C study [11]. Besides, the different median BMI values between Chinese and Caucasians may account for the variance in *β*-cell functions. BMI of subjects in this study and Taiwanese study was 18.36 kg/m^2^ and 14.6 kg/m^2^, respectively, and both were much lower than that of Caucasians. It had been found that type 2 diabetes patients with higher BMI value maintained better *β*-cell function than those with lower value. Whereas the correlation between T1DM and BMI value is still not established. This study did not found any correlation between *β*-cell function and BMI value, similar to the results of Greenbaum et al.'s study [5]. Another assumption was the association of autoantibodies and human leukocyte antigen (HLA) genotypes. Our study did not find the relationship between the failure of *β*-cell function and the numbers of autoantibodies, and the association of autoantibodies or HLA genotypes with *β*-cell function was not the point we focus on because of the declined antibody titer in the process of the disease. Maybe, the immune-mediated mechanism was not as important as that in Caucasians on account of the low prevalence of autoantibodies in Chinese T1DM patients, as reported previously [22, 23].

Age at diagnosis as the strongest predictor for the residual *β*-cell function was a major finding in our study, consistent with other studies [5, 7, 24]. We could find reasonable explanations from embryo physiology to interpret this phenomenon. The replication of *β*-cell during infancy plays a major role in *β*-cell mass up to adult levels. The *β*-cell mass reaches an adult level at the age of 5 years due to it grows rapidly in nondiabetic childhood [25]. Therefore, diabetic children with age at onset ≤ 5 years lost huge numbers of *β*-cells perpetually, which quantified by lower detectable C-peptide level.

The study also found that the younger onset age of T1DM, the faster failure speed of the *β*-cell function. Furthermore, this inevitable decline of stimulated C-peptide followed the log-liner falling pattern in patients with duration < 5 years and obviously slowed down over 5 years. This result was similar to Barker et al.'s finding [7]. This log-liner falling pattern indicated that the islet function failure was the consequence of the loss of islet cells mass, not the deterioration of the quality. The clinical implication of this founding was that the intervention therapy of pancreatic *β*-cell, for instance, immunotherapy should start at the earliest practicable opportunity in the course of T1DM.

Another interesting finding was that the continual failure of *β*-cell function was not obvious in patients with long duration (over 10 years). Even some patients showed a slight recovery of *β*-cell function, which was more common in patients with older onset age. Joslin 50-year medalist study demonstrated that T1DM patients with chronic duration preserved persistence and function of insulin-producing pancreatic cells, endogenous clinical insulin secretion, and sustained positive insulin cells in the pancreatic biopsy [4]. Both studies indicated that residual *β*-cells in T1DM patients with chronic duration were in a steady state of cellular apoptosis and proliferation. The mechanism of the islet function regeneration is still unclear. A recent report from nature demonstrated that *α*-cells could differentiate into *β*-cells in *β*-cells depleted mouse models after prolonged duration (10 months) of diabetes [26]. It is likely to provide a new research direction of pancreatic cell function with long duration.

The relationship between blood glucose and the *β*-cell function was controversial. This study showed that HbA1c and stimulated C-peptide level were well correlated in subjects with duration < 5 years. Furthermore, ROC analysis found that the cut-off point of stimulated C-peptide level was 0.615 pmol/mL, using HbA1c threshold value 7% as outcome variable. Coincidentally, DCCT data demonstrated that patients with preserved C-peptide, defined as stimulated C − peptide level > 0.6 pmol/mL, had superior clinical outcomes, including less hypoglycemia and retinopathy, than those with lower C-peptide [3]. This finding indicated that patients with stimulated C − peptide level ≥ 0.615 pmol/mL more tended to reach the target of HbA1c at the beginning, and this level of stimulated C-peptide may be considered as an expected target of immunological therapies or stem cell therapies in the future.

There was no significant correlation between blood glucose level and stimulated C-peptide level in subjects with duration >5 years. It was likely that most of the patients with long duration had extremely low C-peptide levels, which had little effects on glucose metabolism. Coincidently, the DCCT showed intensive therapy group preserved residual *β*-cell function significantly better than the conventional therapy group in the first 4 years of follow-up, but there were no differences between the two groups in stimulated C-peptide level at the DCCT study fifth and sixth year [2]. There were no explanations for this result from DCCT; here, our result suggested that *β*-cell function in patients with the diabetes duration >5 years likely had poor correlation with blood glucose.

Many previous researches were intervention studies, while interventions would influence the *β*-cell function in the natural process [27–29]. This research was a follow-up study based on the 3C study without special interventions on the *β*-cell function, so we considered the results as objective. Furthermore, different studies used different sampling methods for C-peptide determinations, such as stimulated C-peptide [3, 5], nonstimulated fasting C-peptide [7, 24], or random C-peptide [30], and provided different results. In previous studies, stimulated C-peptide had been proved as the best possible way for estimating residual *β*-cell function. In contrast to stimulated C-peptide, there was limited change in fasting C-peptide during follow-up for several years [3, 5, 31, 32]. That was the reason we used stimulated C-peptide to estimate residual *β*-cell function in this study.

Neither DCCT nor other studies could prove that intensive glycemic control would prevent the pancreatic *β*-cell function from decline in T1DM patients. Although the failure of *β*-cell function was inevitable in most patients with a duration of over 5 years, it was obvious that preserved *β*-cell function could bring significant clinical benefits. Further studies aiming at protecting *β*-cell function in diabetic patients within the first 5 years are urgently needed.

## Figures and Tables

**Table 1:** Epidemiologic and clinical characteristics of all participants in this study.

Variable	Total cohort	Patients with residual *β*-cell function	Patients without residual *β*-cell function	*P* value
Gender (female/male)	101/100	38/36	63/64	0.928
Age (years)	24.77 (15.30, 33.50)	24.29 (12.30, 34.70)	25.10 (15.5, 33.6)	0.870
DKA at diagnosis				
DKA	88	26	62	0.158
Ketosis	35	14	21	
None	78	34	44	
BMI (kg/m^2^)	18.36 ± 4.73	18.55 ± 3.83	18.17 ± 4.92	0.766
Duration of T1DM (years)	5.12 (1.30, 6.60)	3.85 (1.00, 5.85)	5.38 (1.8, 6.8)	0.049
Age at diagnosis (years)	18.72 (11.00, 27.50)	22.95 (12.05, 29.55)	16.36 (9.7, 25.3)	0.002
Presence of family history of diabetes	66	25	41	0.836
Hypoglycemia by 100 patient years	50.44	48.36	61.36	0.535
Ketosis (including DKA) by 100 patient years	32.25	30.95	32.70	0.643
HbA1c (%)	10.04 ± 2.87	8.65 ± 2.57	10.41 ± 2.83	<0.001
HbA1c at diagnosis (%)	10.89 ± 2.74	9.96 ± 2.34	11.03 ± 3.33	0.092
Fasting C-peptide (pmol/mL)	0.14 (0.07, 0.25)	0.20 (0.09, 0.33)	0.08 (0.04, 0.13)	<0.001
Stimulated C-peptide (pmol/mL)	0.29 (0.11, 0.48)	0.57 (0.24, 0.78)	0.10 (0.04, 0.17)	<0.001
Positivity of GAD	95	35	60	0.990
Positivity of CA512	49	18	31	0.946
Positivity of ZnT8	25	12	13	0.315
Methods of insulin administration				
CSII	10	3	7	0.748
4 injection per day	44	14	30	0.483
<4 injection per day	147	57	90	0.410
Insulin dose (IU/kg.day)	0.74 (0.50, 0.94)	0.77 (0.5, 0.91)	0.73 (0.50, 0.94)	0.95
Positivity of diabetic retinopathy (*n*)	22	5	17	0.071
Positivity of proteinuria (*n*)	12	1	11	0.099
Positivity of microalbuminuria (*n*)	54	14	30	0.685
BUN (umol/l)	5.22 ± 1.88	5.38 ± 2.47	5.10 ± 1.31	0.568
CHOL (mmol/l)	4.42 ± 1.03	4.25 ± 0.73	4.55 ± 1.20	0.246
CREAT (mmol/l)	73.88 ± 34.86	79.83 ± 41.10	69.45 ± 29.24	0.253
HDL (mmol/l)	1.41 ± 0.38	1.38 ± 0.36	1.42 ± 0.39	0.723
LDL (mmol/l)	2.17 ± 0.69	2.16 ± 0.57	2.18 ± 0.78	0.932
TRIG (mmol/l)	1.16 ± 0.63	1.31 ± 0.72	1.04 ± 0.54	0.096

DKA: diabetic ketoacidosis; CSII: continuous subcutaneous insulin injection.

**Table 2:** Factors associated with *β*-cell function by COX regression analysis.

Patients with diabetic duration < 5 years			
Variables	Sig.	OR	95.0% CI for OR
Mean HbA1c	<0.001	1.322	1.225-1.555
Age at diagnosis	0.467	0.952	0.917-0.987
Diabetic duration	0.011	1.152	1.032-1.285

Patients with diabetic duration > 5 years			
Variables	Sig.	OR	95.0% CI for OR
Age at diagnosis	<0.001	0.945	0.916-0.975

## Data Availability

The data used to support the findings of this study are available from the corresponding author upon request.
