# Corrigendum to “Residual *β*-Cell Function in Type 1 Diabetes Followed for 2 Years after 3C Study”

**DOI:** 10.1155/2021/9830928

**Published:** 2021-10-31

**Authors:** Kun Lin, Xiaoping Yang, Yixi Wu, Shuru Chen, Qiong Zeng

**Affiliations:** ^1^Department of Endocrinology, The First Affiliated Hospital of Shantou University Medical College, Shantou, China; ^2^Shenzhen Huada Gene Technology Service Co., Ltd., Shenzhen, China; ^3^Department of Neurology, The First Affiliated Hospital of Shantou University Medical College, Shantou, China

In the article titled “Residual *β*-Cell Function in Type 1 Diabetes Followed for 2 Years after 3C Study” [1], Dr. Kun Lin was incorrectly listed as the corresponding author. The corresponding author is Dr. Qiong Zeng, as shown above.
